# CFD Study of a Tunnel-Ventilated Compost-Bedded Pack Barn Integrating an Evaporative Pad Cooling System

**DOI:** 10.3390/ani12141776

**Published:** 2022-07-11

**Authors:** Felipe Andrés Obando Vega, Ana Paola Montoya Ríos, Jairo Alexander Osorio Saraz, Rafaella Resende Andrade, Flávio Alves Damasceno, Matteo Barbari

**Affiliations:** 1 Facultad de Ciencias Agrarias, Universidad Nacional de Colombia, Carrera 65 N^∘^ 59A-110, Medellín 050034, Colombia; apmontoy@unal.edu.co (A.P.M.R.); aosorio@unal.edu.co (J.A.O.S.); 2 Department of Agricultural Engineering, Federal University of Viçosa, Av. Peter Henry Rolfs, University Campus of Viçosa, Viçosa 36570-900, Brazil; rafaella.andrade@ufv.br; 3 Departamento de Engenharia, Universidade Federal de Lavras, Rua Prof. Edmir Sá Santos, University Campus, Lavras 37200-900, Brazil; flavio.damasceno@ufla.br; 4 Department of Agriculture, Food, Environment and Forestry (DAGRI), University of Florence, P. le Delle Cascine, 18, 50144 Firenze, Italy; matteo.barbari@unifi.it

**Keywords:** compost-bedded pack barn, tunnel-ventilated, animal welfare, thermal comfort, dairy cattle, ETIC, CFD, evaporative cooling

## Abstract

**Simple Summary:**

It is known that improving the welfare of cows increases dairy productivity. A compost-bedded pack barn equipped with evaporative cooling pads to regulate the inside environmental conditions of dairy seems to be a synergic combination to improve substantial the welfare in dairy facilities. However, there is a lack of information about both techniques working together. A computational model of a tunnel-ventilated compost-bedded pack barn with an evaporative pad cooling system was developed to know the spatial distribution of temperature, relative humidity and velocity of the air inside the barn. These variables allowed us to compute a thermal stress index for dairy cattle to identify the geometric characteristics and operative conditions of the evaporative pad cooling that provide the best environmental conditions inside the barn according to the outside environmental conditions.

**Abstract:**

Evaporative cooling is one of the most efficient techniques to reduce heat stress in cows in agricultural facilities. Additionally, compost-bedded pack barn has been shown to improve the welfare and production of cows. Two techniques were combined and analysed by developing a computational fluid dynamics (CFD) model of a tunnel-ventilated compost-bedded packed barn that integrated the heat and airflow dynamics of an evaporative pad cooling system. This allowed us to study the distribution of dry-bulb temperature, relative humidity and airflow velocity inside the barn based on the external environmental conditions, thickness of the pad, water temperature and specific manufacturer characteristics of the pad, providing optimal cooling pad location, size and operating conditions in the barn. Employing experimental data the CFD model was validated showing good agreement. The Equivalent Temperature Index for dairy Cattle (ETIC) was used to determine the level of stress of the cows considering the airflow velocity. It was found a moderate stress due to high relative humidity and low airflow velocity. From the predicted results, it was recommended to increase the airflow velocity above 3 m s${}^{-1}$ when simultaneously the external dry-bulb temperature and relative humidity exceed 30 °C and 55%, respectively, simultaneously. Additionally, installation of baffles at the pad outlet to drive the airflow to the floor was suggested to improve the drying of the compost-bedded closed to the pads, where a low airflow velocity region was established.

## 1. Introduction

Compost-bedded pack barn (CBP) is an animal housing system that provides a resting area for dairy cows free of stalls or partitions, generating a healthy and comfortable open environment with a floor surface made of absorbent and biodegradable material as sawdust or straw [1,2]. CBP has received attention due to the increase in production, comfort and health of the animals, in comparison with traditional freestall systems when properly maintained CBP [3,4,5,6]. Scientific literature had been focused on determining the airflow distribution inside dairy cow barns with natural [7,8,9] and mechanical ventilation [10,11,12] for freestall and CBP systems, but none involve the distribution of the dry-bulb temperature ($T_{db}$) and relative humidity (ϕ).

On the other hand, evaporative cooling has been shown to be one of the most efficient technique to reduce the thermal stress on cows through foggers, misters, sprinklers or baths, located in specific zones of the barns [13,14,15]. However, the benefits of these methods last as long as the skin of the animal remains wet and it depends on the coverage of the systems. Meanwhile, tunnel-ventilated barns with evaporative pad cooling provides micro-environmental conditions that improve animal welfare inside the facility, increase the milk production and minimizing the addition excess of water into the cow’s environment [16,17,18,19,20].

The use of evaporative pad cooling in tunnel-ventilated barns in tropical and sub-tropical locations, which are characterized by high environmental ϕ levels, is controversial due that this variable reaches high levels inside the facilities. However, it has been proved that evaporative pad cooling provides comfort levels higher than those obtained on natural and mechanical ventilated barns, alleviating the thermal stress on cows when it is needed [18]. Even though some research studies do not recommend the use of evaporative cooling techniques with CBP barns due to the relation of high levels of ϕ inside the facility with the poor drying rate of the bed [3]. However, the benefits of *CBP* and evaporative pad cooling systems on cow welfare has promoted the use of both systems together, using the air velocity to partially mitigate the impact of high relative humidity [3,17]. For this reason, false ceiling and baffles are usually located on the roof to redirect and concentrate the airflow over the cows, increasing the cooling effect [8,21] with airflow velocities above 3 m s${}^{-1}$ [8] which is the recommended value for tunnel-ventilated barns [17,22].

The Temperature–Humidity Index (*THI*) has been commonly used to quantify the thermal stress level of the animals, linking the dry-bulb temperature and relative humidity [23,24,25]. Wang et al. [26] recently proposed the Equivalent Temperature Index for dairy Cattle (ETIC) as substituted of *THI* (Equation (1)), considering the environment dry-bulb temperature ($T_{db}$), relative humidity (ϕ), air velocity (*u*) and solar radiation effects (*G*) in the computation of the index. The heat stress thresholds were characterized as mild (18 °C ≤ ETIC < 20 °C), moderate (20 °C ≤ ETIC < 25 °C), severe (25 °C ≤ ETIC < 31 °C) and emergency (ETIC ≥ 31 °C).

(1) $$\mathrm{ETIC}=T_{db}-0.038T\left(100-\phi \right)-0.1173u^{0.707}\left(39.2-T_{db}\right)+1.86\times 10^{-4}T_{db}G$$

A lot of studies are focused on the impact of microclimatic conditions over lactating dairy cows and their spatial distribution freestyle barns facilities [27,28,29,30]. However, there is scarce information about the distribution of the dry-bulb temperature and relative humidity that could lead to improvements in CBP design. Thus, this research study serves as a first approximation of modeling and simulation of the thermal distribution inside a tunnel-ventilated CBP dairy barn with evaporative pad cooling system located in a tropical environment, using computer fluid dynamics (CFD) simulations. This computer tool has been widely shown to be an accurate complement to experimental field tests, leading to the analysis of a wide range of operative conditions [31].

## 2. Materials and Methods

### 2.1. Experimental Tests in Tunnel-Ventilated Compost-Bedded Pack Barn

**Figure 1:**
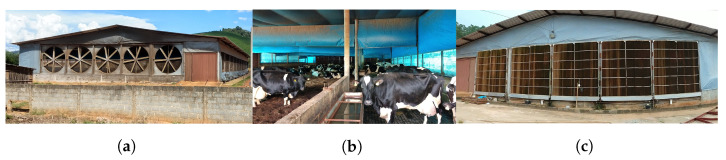
Tunnel-ventilated compost-bedded pack barn. (**a**)—Fan exhaust. (**b**)—Inside. (**c**)—Evaporative cooling pads.

Experimental tests were made in a tunnel-ventilated compost-bedded pack barn with 80 Holstein cows in the lactation cycle (about 600 kg and 11 m${}^{2}$ per cow) and equipped with an evaporative pad cooling system (Figure 1) during the dry season in December of 2018. The facility has an external length of 55 m, width of 26.4 m and height of 5 m at the ridge and is located in the city of Viçosa, Minas Gerais, Brazil (latitude 20°46′41″ S, longitude 42°48′57″ W and altitude 657 m). It has a drive-through feed platform of 55 × 4 m${}^{2}$, a feed alley of 55 × 4 m${}^{2}$ with four drinkers, a compost-bedded pack of wood shavings and coffee husk of 55.0 × 16 m${}^{2}$, and a service alley of 55 × 2.4 m${}^{2}$. The floor of the three alleys is made of concrete and the feed alley and the compost bedded is separated with 1.2 m high concrete walls. The building has metallic roof with a plastic ceiling of polypropylene fabric at 4.4 m of height, lateral walls of 1 m and lateral sides cover with polypropylene fabric curtains. Five baffles of polypropylene fabric are located at 3 m above the floor and separated 11 m from each other. In addition, a polypropylene fabric curtain from 2.2 m above the floor to the ceiling is used to separate the drive-through feed alley from the other areas. These are used to concentrate the airflow over the cows. The compost-bedded tilling is performed with a modified cultivator on a small tractor when cows are milked, at 06:00 and 17:00 h every day.

Five exhaust fans (BigFan^®^, 3.5 m of diameter, six blades, airflow of 150,000 m${}^{3}$ h${}^{-1}$ and 2.0 hp, Airway-Engenharia e Equipamentos Ltda, Caxias do Sul, Rio Grande do Sul, Brazil) force the air to pass through five cellulose pads each one of 3.6 × 3.6 m ($45^{\circ }\times 15^{\circ }$ angles, CIABRAPE, Empresa Brasileira de Papéis Especiais, Campinas, Brazil) continually wetted and oppositely located at a distance of 55 m. The airflow through the feed alley was driven meanly by one exhaust fan and one cooling pad, while for the compost bedded area four exhaust fans and four cooling pads were used. The exhaust fans stayed on all the time while the evaporative cooling system was turned on only when a temperature and relative humidity sensor located in the middle of the facility measured a temperature above 21 °C and a relative humidity below 75%.

**Figure 2:**
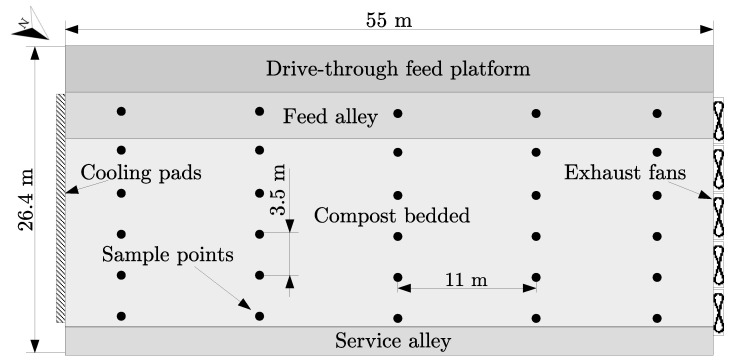
Dimensions and measured points inside the closed compost dairy barn building.

Twenty five measurements points were defined in the compost bedded area and five points in the feed alley area at a high of 2 m above the floor, the sensors were arranged as shown in the Figure 2. Dry-bulb temperature and relative humidity were measured using digital sensors (DHT22; range measurements: 0 to 100% for relative humidity and −40 to 80 °C for dry-bulb temperature; accuracy: ±2% and ±0.5 °C; Aosong Electronics Co., Ltd, Guangzhou, China). Thermal conditions outside the facility were also measured using the same type of sensors inside a meteorology cover. Several Arduino Mega 2560 Rev. 3 open-source electronic developed platform (https://www.arduino.cc/, accessed on 3 July 2022) were used as dataloggers to collect the data, with up to four sensors per device.

The air velocity inside the barn was measured manually using a hot-wired anemometer (INSTRUTHERM^®^, TAFR-180 model with an accuracy of ±0.1 m s${}^{-1}$, Instrumentos de Medição Ltda, São Paulo, Brazil) in the measurement points previously defined. It was found that the variation of the air velocity for each point was near to the sensor error, and consequently an average air velocity was considered.

### 2.2. CFD Model of Evaporative Cooling Pad

A pad section of 0.5 m of height, 0.01 m of width and 0.1 m of thickness was meshed, modelled and simulated. The commercial ANSYS CFX^®^ (ANSYS Academic Student Released 2020 R1, ANSYS Inc., Canonsburg, PA, USA) CFD software was used to solve the equations of conservation of mass, momentum, turbulence, heat and mass transfer for incompressible and turbulent fluid in steady state, through a fully-couple finite volume solver. The turbulence k-ϵ model with scalable wall function was employed to simulate the turbulence. From the ANSYS CFX^®^ CFD software, a solver advection scheme option of high resolution, corresponding to a second order upwind scheme, was selected. As convergence criteria the RMS residual type was used with a RMS residual target of $1\times 10^{-4}$ for all models.

**Figure 3:**
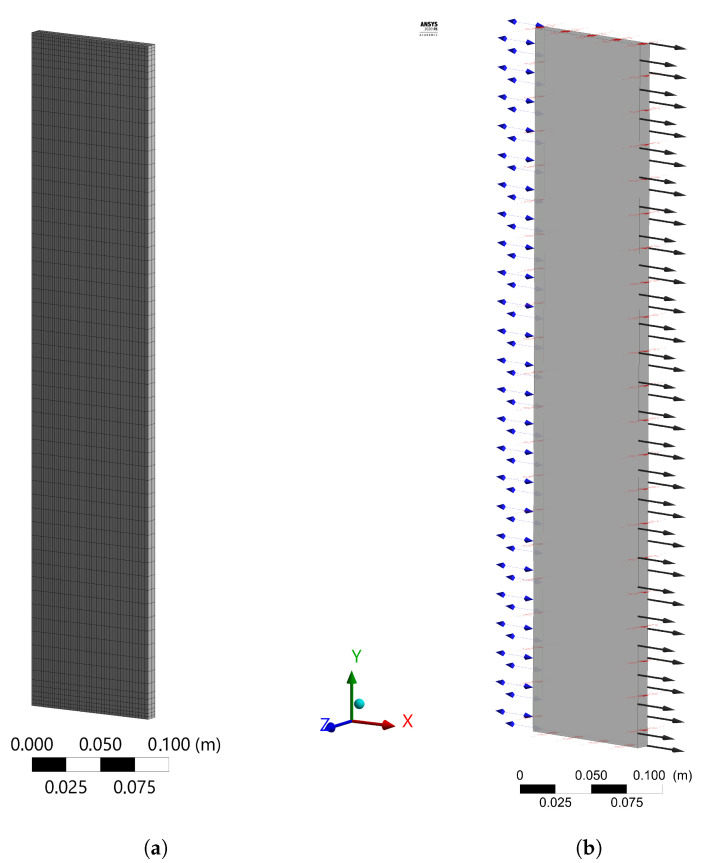
Meshing (**a**) and boundaries setup (**b**) of the pad model in the ANSYS CFX^®^ software. The inlet and outlet of the pad are represented with double and single arrows, respectively.

A structured mesh with linear order elements was used to meshing the pad model. Element sizes of 0.025 and 0.01 m were defined along the height and width of the pad section, respectively. Several refinements of the mesh in the flow direction (thickness) were compared. Five inflation layers with a first layer 0.002 m and 1.2 of growth rate were defined in the top and bottom regions of the pad. The pad meshing for 50 divisions in the flow direction (*x*-axis) is shown in Figure 3a.

The cellulose pad was modelled as a homogeneous isotropic porous medium with a porosity of 0.94, permeability of 1.4853 × 10${}^{-6}$ m${}^{-2}$, coefficient of Forchheimer of 22.2652 m${}^{-1}$ corresponding to a water flow across the pad of 6.2 L min${}^{-1}$ m${}^{-1}$, and specific surface area (ζ) of 297.16 m${}^{-1}$ [32]. The inlet air was considered as an ideal gas mixture of dry air and water vapour modelled using the species transport model, where the kinematic diffusivity of water vapour in dry air ($D_{AB}$) was considered as a function of dry-bulb temperature ($T_{db}$ [°C]) and the air pressure (*P* [Pa]) as can be seen in Equation (2) [33].

(2) $$D_{AB}=\frac{926\times 10^{-6}}{P}\frac{{\left(T_{db}+273.15\right)}^{2.5}}{T_{db}+518.15}\;[m{}^{2}\;s{}^{-1}]$$

The inlet boundary condition of the pad was established as an opening of entrainment type with 93,620 Pa as relative pressure, and the turbulence boundary condition was set as zero gradient turbulence. The psychometric properties of the air at inlet of the cooling pad and the mass fraction of water vapour were established according to the experimental measurements. The boundary at the pad output was defined as outlet with a normal speed of 1.2 m s${}^{-1}$ according to the experimental measurements. Wall boundary types were selected for the upper and lower surfaces with adiabatic thermal conditions and the lateral surfaces were defined as symmetry boundary types. The procedure to convert the relative humidity to mass fraction of the water vapour, required at the inlet boundary condition, is presented in the Appendix A. Those boundaries are shown by arrows in the Figure 3b. The inlet and outlet boundaries conditions are represented with double and single arrows, respectively.

The temperature of the surface of the porous medium ($T_{s}$) was supposed equal to the inlet air wet-bulb temperature due to the adiabatic cooling process existing through the wetted pads (equilibrium temperature between the air and the water flow) [34]. Heat transfer between the pad surface and the wet air was modelled using the thermal energy model. The convective heat transfer coefficient ($h_{H}$) was computed using the correlation of Hilper modified for cellulose cooling pads (3) [32] and was included in the porosity settings of the domain of the pads to include the dynamics of the heat transfer between the fluid and the solid (pad).

(3) hH=1.042leL0.426Re0.617Pr13

where *Re* (Equation (A6)), *Pr* (Equation (A7)) and *Sc* (Equation (A8)) were Reynolds, Prantl and Schmidt dimensional numbers, respectively [35]; $l_{e}$ is the specific length, compute as the inverse of ζ, *L* is the thickness of the cooling pad, *k* is the thermal conductivity of wet air (Equation (A11)) and $D_{AB}$ is the mass diffusion coefficient.

The convective mass transfer coefficient ($h_{M}$) that was computed using the correlation of Hilper modified for cooling pads (Equation (4)) [32] was used to predict the water evaporated rate (${\dot{m}}_{e}$, Equation (5)) from the wetted pad surface. This value was added as a mass source to the equation of species transport solved by the CFD software.

(4) hM=0.385leL0.513Re0.724Sc13

(5) $${\dot{m}}_{e}=h_{M}A_{s}\left(\rho_{v_{@T_{s}}}-\rho_{v_{@T_{f}}}\right)$$

where $A_{s}$ was the wetted surface area computed as the product of the specific surface area by the volume of the pad simulated, $\rho_{v_{@T_{s}}}$ and $\rho_{v_{@T_{f}}}$ were the density of the water vapour (Equation (A14)) at the surface temperature of the pad ($T_{s}$) and the free flow air temperature ($T_{f}$), respectively.

To evaluate the goodness of the CFD model, 50 random measurements from the experimental tests [32] were selected and compared with the CFD model results by means of the Root Mean Square Error (RMSE), the Wilcoxon rank sum test (*ranksum* function, MATLAB^®^ Released 2018a, The MathWorks, Inc., Tokyo, Japan) and regression analysis. The measurements and the predicted dry-bulb temperatures and relative humidity were compared.

### 2.3. CFD Model of Tunnel-Ventilated Compost-Bedded Pack Barn

**Figure 4:**
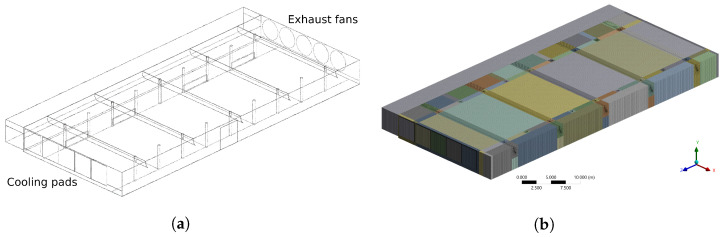
Model (**a**) and meshing (**b**) of the dairy cattle barn in the ANSYS CFX^®^ software.

The airflow domain of the CBP barn, including the evaporative pads, was modelled using SOLIDWORKS^®^ CAD software (released 2018, Dassault Systèmes SolidWorks Corporation, Waltham, MA, USA) as shown in the Figure 4a. This model was imported into the ANSYS^®^ CFX software (ANSYS Inc., released 2019 R2) to meshing and simulation. A multiblock hybrid mesh using structured mesh was employed to reduce the number of elements [36]. Adaptive sizing was used with a resolution of three, to reproduce the airflow around the roof curtains and columns geometry properly. A mesh sensibility analysis was performed with coarse, medium and fine mesh sizes to select the one that provides an adequate balance between computational accuracy and efficiency. In Figure 4b a medium mesh sizes is shown.

The ANSYS CFX^®^ solver was used to simulate the facility. As convergence criteria the RMS residual type was used with a RMS residual target of $1\times 10^{-4}$ for all the models. The fluid was considered as an ideal gas mixture of dry air and water vapour modelled using the species transport model as defined before. To model the heat transfer between both fluids the thermal energy model was used. The turbulent flow was modelled by the k-ϵ model with scalable wall function. From the ANSYS CFX^®^ CFD software, a solver advection scheme option of high resolution, corresponding to a second order upwind scheme, was selected. The cooling pads and the exhaust fans were include in the computational domain of the barn. For this, the pads were modelled using the same procedure described previously. The boundary condition of the exhaust fans were considered as outlet.

Thermographic camera (INSTRUTHERM^®^, ITTMV-100, accuracy of ±2% of measurement, Instrumentos de Medição Ltda, São Paulo, Brazil) was used to measure the temperatures inside the building (Appendix D). Due that the temperature of the surface of the compost bed increases approaching to the exhaust fans, it was considered that it changes proportionally to the distance from the pads (*z*), from 25.4 °C at the pads to 30 °C at the exhaust fans. Table 1 summarizes the boundaries conditions used in the CFD model.

**Figure 5:**
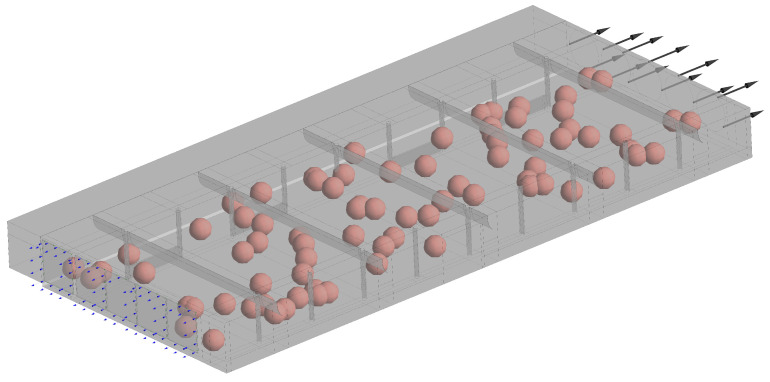
Setup model in the ANSYS CFX^®^ CFD software. The spheres represent the source points of convective heat loss from the cows, without mass, without presenting any resistance in the air flow and spatially random distributed (Appendix C). The red arrows represent the interface between the blocks meshed. The blue arrows are the opening boundary at the pad inlet and the black arrows represent the boundary condition of the exhaust fans.

The convective heat loss from the cows was simulated as energy source points without mass and without presenting any resistance in the air flow. They were located randomly around the compost bedded and the feed alley at 1.2 m height (Figure 5, Appendix C). For this, the correlation suggested by Wang et al. [37] for horizontal airflow was used to compute the convective heat transfer coefficient from the Nusselt dimensionless number as $Nu_{c}=0.082Re_{c}^{0.707}$, where Reynolds number ($Re_{c}=\frac{ul_{c}}{\nu }$) was computed in terms of the airflow velocity (*u*, [m s${}^{-1}$]), the kinematic viscosity (ν, [m${}^{2}$ s${}^{-1}$]) and the characteristic length for the cow ($l_{c}$, [m]), which was considered equal to the heart girth (HG, [m]) and body weight (BW, [kg]) of the cow as $l_{c}=HG=\frac{BW+324.52}{417.5}$ [38,39].

The convective heat transfer coefficient was then obtained as $h_{H_{c}}=\frac{Nu_{c}k}{l_{c}}$ where *k* is the thermal conductivity of the air (Equation (A11)). The convective heat loss from the cow was computed as $Q_{c}=h_{H_{c}}SA\left(T_{db}-T_{b}\right)$ [35]. Where SA is the skin surface area of the cow, calculated in terms of the body weight of the cow as $SA=0.14BW^{0.57}$ [40], $T_{b}$ is the temperature of the surface of the cow and $T_{db}$ is the dry-bulb temperature of the airflow.

Additionally, the dry-bulb temperature and the mass fraction of the water vapour (see Appendix A) was defined as the thermal conditions of the inlet air in the opening boundary. A reference pressure of 95,320 Pa was used in the CFD model.

To simulate the water evaporated from the compost bedded, the evaporation rate found by Bjerg and Klaas [41] ($2.2557\times 10^{-4}$ kg d${}^{-1}$ per cow) was used. For this, it was divided by the area available for each cow (11 m${}^{2}$), obtaining a water vapour mass flux of $2.0506\times 10^{-5}$ kg m${}^{-2}$ s${}^{-1}$. This value was added as a water vapour source to the equation of species transport solved by the CFD software.

The ETIC (Equation (1)) was used to analyse the thermal comfort inside the facility, considering the solar radiation (*G*) equal to zero.

## 3. Results

### 3.1. CFD Modelling of Evaporative Cooling Pad

The analysis of mesh independence of the cooling pad model showed that when the thickness of the pad was divide in 50 Sections (0.002 m element size), 0.57 °C and 1.55% were obtained for RMSE values of the dry-bulb temperature and relative humidity of the air at the pad outlet. When the number of divisions was increased up to 300, a difference of 0.03 °C and 0.49% with respect to 50 division were presented, not significant improvement in results was obtained at the cost of an increase in the computational time. Then, 50 division were selected as a properly value to meshing the thickness of the cooling pad.

Table 2 shows the statistics of the inlet and outlet psychometric properties of air for the 50 random experimental tests used to validated the goodness of the CFD model.

**Figure 6:**
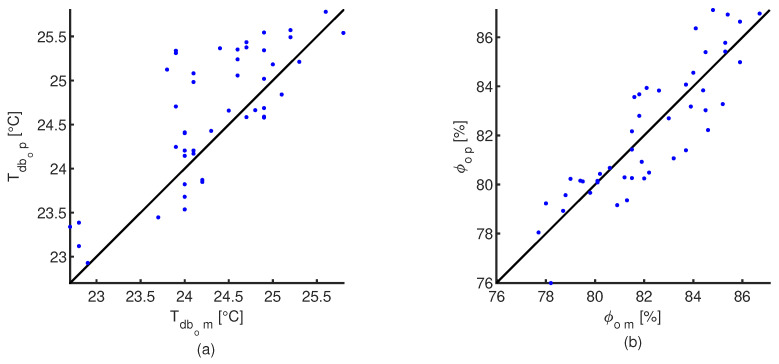
Relationship between the thermal conditions at pad outlet predicted with the CFD model (*p*) and experimental measurements (*m*). (**a**) —Outlet air dry-bulb temperature ($T_{db_{o}}$). (**b**)—Outlet air relative humidity ($\phi_{o}$). Solid black line corresponds to the equality line.

**Figure 7:**
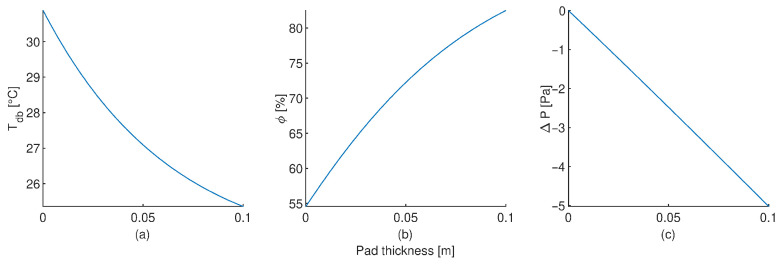
CFD predictions of the dry-bulb temperature (**a**), relative humidity (**b**) and pressure drop (**c**) changes inside the cellulose pad. For 30.9 °C, 54.5% and 1.2 m s${}^{-1}$ of dry-bulb temperature, relative humidity and velocity of the air at inlet pad, respectively.

A comparison between the predicted values of dry-bulb temperature and relative humidity at the pad outlet with the proposed CFD cooling pad model and the measure values is shown in Figure 6. A equality line (black solid line) is also plotted for comparison purposes. Wilcoxon sum rank statistical tests were apply to both dry-bulb temperature and relative humidity sets of data, obtained *p*-values of 0.054 and 0.920, respectively. This indicates that there is not enough statistical evidence to conclude that the measure of central tendency of the predicted values are different to the experimental measurements (α=0.05). Figure 7 shows how the dry-bulb temperature, relative humidity and pressure drop, change inside the simulated cooling pad. The output provided for the CFD model for the dry-bulb temperature, relative humidity and pressure drop are shown in Appendix B for an air velocity of 1.2 m s${}^{-1}$ and external environmental conditions of 30.9 °C and 54.5%.

### 3.2. CFD Modelling of Tunnel-Ventilate Compost-Bedded Pack Barns

#### 3.2.1. CFD Model Validation

Four mesh sizes of 1,048,893, 2,675,770, 4,655,442 and 10,236,714 nodes, defined as coarse, medium, fine${}_{1}$ and fine${}_{2}$, respectively, were evaluated to determine if the results are independent from the meshing process. For the average outside environmental conditions obtained between 10:00 and 11:00 h (27.5 °C, 72.8% and 2.0 m s${}^{-1}$) simulations were performed. When the fine${}_{2}$ mesh was employed in the simulations, 1.06 °C and 11.9% were obtained for RMSE computed between the average measurements and the CFD predicted values for the dry-bulb temperature and relative humidity, respectively. A variation up to 0.02 °C and 0.023% were obtained for the RMSE values when the coarse mesh was used. This low variability allows to conclude that the results are not influenced by the mesh sizes analysed. The medium mesh size was selected for the following simulations to obtain a suitable representation of the distribution of the variables of interest inside the building. A detailed description of the characteristics of the four mesh sizes evaluated is shown in Table 3.

**Figure 8:**
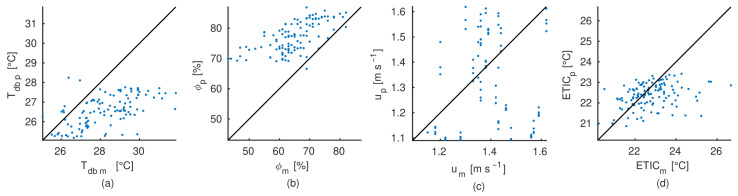
Regression analysis of the experimental measurements (*m*) and CFD predicted (*p*) conditions inside the tunnel-ventilated compost packed dairy barn using the CFD model developed. (**a**)—Dry-bulb temperature ($T_{db}$ [°C]). (**b**)—Relative humidity (ϕ [%]). (**c**)—Airflow velocity (*u* [m s${}^{-1}$]). Solid black line corresponds to the equality line. (**d**)—Equivalent Temperature Index for dairy Cattle (ETIC [°C]). Solid black line corresponds to the equality line.

The relationship between the measured and predicted values for the dry-bulb temperature, relative humidity, air velocity and ETIC of the air inside the facility are shown in Figure 8. Biased relationships were observed for the dry-bulb temperature and relative humidity. The CFD model was unable to reach the high levels of dry-bulb temperature measured and consequently, the relative humidity could not decrease, remaining in levels above 70%. According to the statistics show in Table 4, the average mean bias deviations were around 1.5 °C and up to 11% for the dry-bulb temperature and relative humidity, respectively. Standard deviation values confirm that the variation of measurements are higher that the predicted values. Despite of this, the ETIC relationship show less deviation from the equality line, with an average mean bias of 0.26 °C and a prediction variability less than 0.6 °C. A random distribution around equality line was observed for the air velocity relationship, the CFD model predictions show a mean and variability similar to the measurements. The variability between the experimental measurements and the CFD model predictions was quantified using the average RMSE, obtaining 2.0 °C, 13.4%, 0.24 m s${}^{-1}$ and 1.1 °C for $T_{db}$, ϕ, *u* and ETIC, respectively. Those values represent relative differences of 6.1%, 19.7%, 3.6% and 14.6%, for each of the simulated variables.

**Figure 9:**
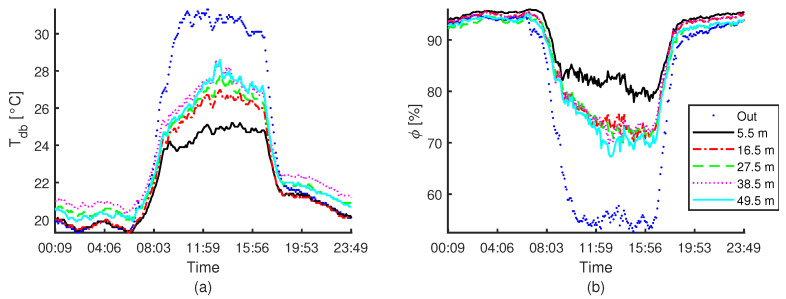
Dry-bulb temperature ($T_{db}$, (**a**)) and relative humidity (ϕ, (**b**)) measurement for one day of experimentation for the outside conditions and inside measurements along the centre of the compost bed. The distance between sensors and the cooling pads is indicate.

Outside and inside dry-bulb temperature ($T_{db}$) and relative humidity (ϕ) along the centre of the compost bed for one day of experimentation are shown in Figure 9. Since the activation of the evaporative pad cooling system (09:00), a significant gradient of the thermal conditions is present from the pad outlet (measured at 5.5 m) up to a distance of 16.5 m, where is located the next sensor. No significant changes in the thermal conditions were observed from this point to the end of the barn (measured at 49.5 m). The gradient increment along the day, even if the external dry-bulb temperature decreased, and is more significant for the sensors located closed to the exhaust fans. The maximum gradient is presented around 14:00 h.

#### 3.2.2. CFD Model Predictions

**Figure 10:**
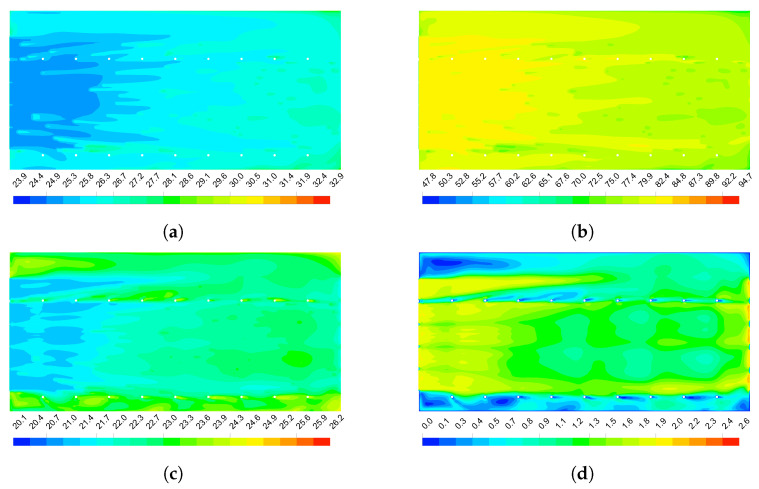
Distribution of the air thermal variables analysed inside the tunnel-ventilated compost-packed bedded dairy barn 1.6 m above the floor. (**a**)—Dry-bulb temperature (°C). (**b**)—Relative humidity (%). (**c**)—Equivalent Temperature Index for dairy Cattle (ETIC, °C). (**d**)—Air velocity (m s${}^{-1}$). The airflow direction is from left to right.

Figure 10 shows the distributions of the dry-bulb temperature, relative humidity, ETIC and air velocity inside the studied barn at 1.6 m above the floor, for an air velocity at the exhaust fans of 2 m s${}^{-1}$ and outside environmental conditions of 27.5 °C and 72% for dry-bulb temperature and relative humidity. Figure 10a,b show that the dry-bulb temperature and relative humidity follow the observed experimental behaviour, increasing the dry bulb temperature and consequently decreasing the relative humidity from a distance around 17 m from the cooling pads. The ETIC distribution presented in Figure 10c shows a detailed representation of the micro-environmental conditions inside the barn. It is observed that the drive-through feed and service alley present high ETIC values along the barn and that the feed alley increase the ETIC values around the first 30% of the barn. The distribution of the air velocity (Figure 10d) shows that the curtain that divide the drive-through feed alley from the feed alley satisfactory drive most of the cooled air over the feed alley, maintaining in low levels the airflow in the drive-through feed alley. However, it is observed that the increasing in the ETIC values over the feed alley is due to decreasing the airflow velocity for the drinker walls and columns that limit the airflow from the others pads. The service alley shows low airflow velocity because the columns that act as barriers limiting the free airflow.

**Figure 11:**
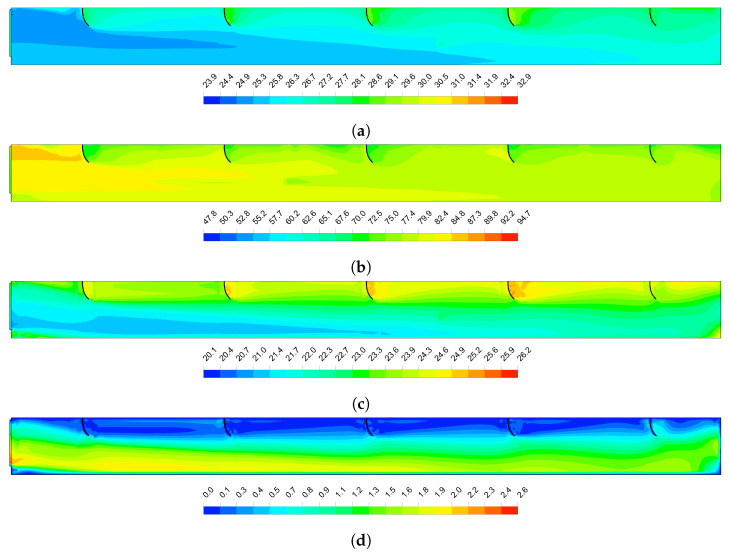
Distribution of air thermal variables analysed along the centre of the tunnel-ventilated compost-packed bedded dairy barn. (**a**)— Dry-bulb temperature (°C). (**b**)—Relative humidity (%). (**c**)—Equivalent Temperature Index for dairy Cattle (ETIC, °C). (**d**)—Air velocity (m s${}^{-1}$). The airflow direction is from left to right.

The distribution of the variables along the centre of the compost-bedded pack shown in Figure 11 complements the aforementioned. The baffles successfully redirects the cooled air to the cow’s level (Figure 11d), maintaining the ETIC values in the thermal comfort zones along the barn (Figure 11c). However, zones of low airflow were created in the volumes between the baffles, increasing the time of contact of the air with the false ceiling leading to an increment of its temperature. The mixture of this hot air and the cooled air from the pads is evident from the second baffle causing that the dry-bulb temperature of the air on the main air stream increase progressively (Figure 11a).

At compost level, the dry-bulb temperature of the air is uniform along the first half of the barn and then progressively increases. A layer of high relative humidity is presented in the first half of the barn at compost level, becoming more evident below the cooling pads (Figure 11a). In this area, a low airflow velocity zone is created, making the dry process of the compost-bedded pack difficult. As can be seen in Figure 11c, this zone extends for almost 3 m in front of the cooling pads.

**Figure 12:**
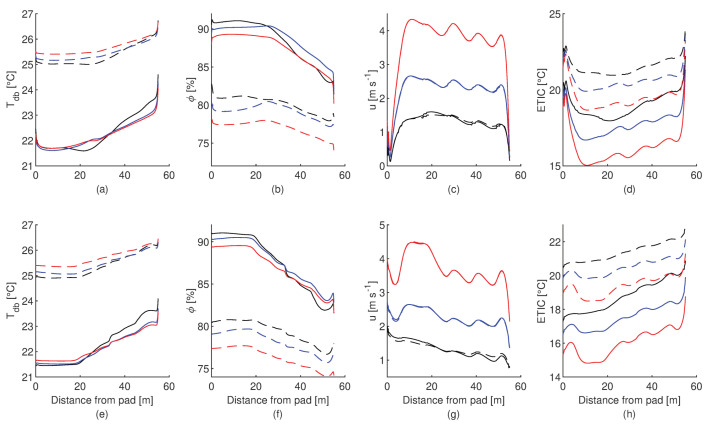
Profile of variation of the dry-bulb temperature ($T_{db}$), relative humidity (ϕ), Equivalent Temperature Index for dairy Cattle (ETIC) and air velocity (*u*) along the centre of the tunnel-ventilated compost-packed bedded dairy barn. At 0.2 m (**a**–**d**) and 1 m (**e**–**h**) above the compost bedded, for two environmental conditions for dry-bulb temperature-relative humidity of 24 °C—75% (solid line) and 30 °C—55% (dashed line), and three airflow velocities across the exhaust fans: 1.5 m s${}^{-1}$ (black line), 3 m s${}^{-1}$ (blue line) and 5 m s${}^{-1}$ (red line).

Two environmental conditions (30 °C—55% and 24 °C—75%) were simulated using airflow velocities of 1.5, 3 and 5 m s${}^{-1}$, to determine the influence of this variable over the analysed variables. Figure 12 shows the profile of the variables along the centre of the barn for each condition simulated at 0.2 and 1 m above the compost bedded. As air velocity increases, the dry bulb temperature rises and the relative humidity decreases at the pad outlet due to the drop of pad cooling efficiency at higher air velocities. In the first 20 m of the barn, the dry-bulb temperature has no significant changes in comparison with the relative humidity at both levels. The behaviour of the dry-bulb temperature and relative humidity for an air velocity of 1 m s${}^{-1}$ is different than for higher air velocities, mainly due to the increase in the contact time of the air with the surroundings that increase the heat transfer rate. At 0.2 m above the compost bed and close to the pads is evident that the airflow has low velocity and reach the free flow velocity approximately 5 m ahead. In addition, this level present the higher ETIC values for all the airflow velocities simulated.

## 4. Discussion

A CFD model of a tunnel-ventilated compost-bedded pack barn that integrate an evaporative pad cooling system model was presented. This is an innovative methodology that allows to simulate the barn using the known external environmental conditions instead of the thermal conditions of the pad outlet, which depends of multiple variables as pad thickness, manufacturer, air velocity, water temperature and input dry-bulb temperature and relative humidity.

The CFD model of the cooling pad proposed, presented good agreement with the experimental as was established for the RMSE values computed. High relative differences in the dry-bulb temperature and relative humidity predictions were obtained in the validation process of the CFD model of the barn, which similar ones were reported by Yao et al. [42]. These differences could be attributed to several factors. First, the CFD model of the pad considered that the entire pad is wetted and adequately airflow across of it. However, in practice, it is possible to have zones of the pad clogged or dry, generating that streams of hot or partially cooled air enter the facility [43]. As the sensors are located close to the pad, narrow streams of air are difficult to detected with one sensor. Second, the accuracy of the sensors employed for measure the relative humidity (±5%) gives a wide margin of uncertainly. Third, variation in the solar radiation along the day was not considering in the model. This changes the walls temperature, as was shown in Figure 9, affecting the temperature inside the barn. Despite of that, the CFD model of the pad is considered that gives an appropriate description of the behaviour of the variables analysed, showing no significant difference between the experimental measurements and predictions, which is in concordance with the experimental results from [16] that report average micro-environmental conditions inside the barn of 28 °C and 84%. Other experimental research studies reported high levels of relative humidity in tunnel-ventilated barn with evaporative pad cooling system [13,17,18,21]. A similar distribution of temperature and the Equivalent Temperature Index for diary Cattle were obtained by [8] who evaluated the baffle positions inside a tunnel-ventilated barn. The average dry bulb temperature difference between external and internal conditions were 2.1 to 5.8 °C, analogue results were obtained experimentally by Smith et al. [18] with 3.1 to 5.2 °C.

The wake distribution of dry-bulb temperature after the source points of heat used to simulate the cows are in agreement with the work of [44] who found an increase in the dry-bulb temperature up to 3 °C after the cows.

As in the CBP systems the cows are widely spaced, it was not considered to represent the zone of animals as a porous medium [11,44] because it would affect the air distribution dramatically. Instead of that, it is recommended to include the cows in the simulated CFD model to obtain a more accurate model in future works.

The CFD model proposed here serves as a tool to improve the design of tunnel-ventilated CBP barns. As was presented, the height at which the cooling pads are installed could affect the compost dry process because of the presence of low airflow velocity zones in front of the pads. Additionally, cows may congregate around this region during heat stress conditions, due to its low levels of ETIC, increasing the manure and urine content of the pack [3]. Thus, to improve the drying of the compost-bedded and its stability, it is recommended to decrease the distance between the floor and the pads during the design of the barn or install baffles at the outlet of the pad that redirect some of the airflow to the compost immediately after the pads in already built installations.

Given the importance of the airflow velocity in this facilities, the inclusion of this variable in a comfort index for dairy cows has contributed to establish satisfactorily the thermal stress levels in ventilated barns. The impact of the dry-bulb temperature, relative humidity and airflow velocity over the thermal comfort of the cows could be analysed in terms of the ETIC index. In this research study, this allows to stablish that the cows in the barn analysed present a moderate level of thermal stress during the day. From the CFD model predictions, it was found that the stress level could be lowered to mild if the airflow velocity increasing above 3 m s${}^{-1}$ when the external dry-bulb temperature and relative humidity exceeds 30 °C and 55%, respectively.

Special attention should be paid to the zones of low airflow velocity that are in contact with the facility walls, as the service alley, because the airflow is susceptible to increase its dry-bulb temperature by the prolonged contact with the walls and increase the inside temperature when it is mixed with the cooled air, increasing the level of thermal stress.

## 5. Conclusions

A novel approach to model a tunnel-ventilated compost-bedded pack barn with evaporative pad cooling systems was presented in this research. This model integrates the behaviour of the fluid-thermal dynamics of the pad cooling with the computational model of the barn, allowing to simulate the facilities using the external environmental conditions instead of suppositions regarding the output conditions of the cooling pads.

The compost-bedded pack barn provides a comfortable and cool place for the cows to lay, but it is require that the air thermal conditions remains below the stress levels to increase the welfare. The CFD model proposed, allowed us to identify a mild stress level in the compost bed area and the influence of the compost-bedded pack over the air conditions of the barn. Additionally, the effectiveness of the baffles and separation curtain used inside the facility was verified and areas of poor ventilation were identified.

Changes required to the airflow at the pad outlet were recognised from the results. Due to the location (high) of the pads, a low ventilation zone was identified in front of them, this area could lead to a high moisture content in the compost and a deactivation of the composting process. Thus, air baffles at low level of the pad outlet are recommended to redirect the airflow to the floor to improve the drying process of the compost bed.

The influence of variations of the airflow velocity through the exhaust fans were studied. Low airflow velocities were associated with increasing the dry-bulb temperature inside the facility. Airflow velocities over 3 m s${}^{-1}$ did not show a significant improvement in the internal thermal condition, however, due to an increase in the heat and mass transfer between the animals and the air, reduce the level of heat stress.

## Figures and Tables

**Table 1:** Boundaries conditions used in the CFD compost dairy barn model.

Location	Boundary Type	Mass and Momentum	Thermal Condition	Value
Roof ceiling	Wall	No slip wall—Smooth wall roughness	Isothermal	34 °C
Lateral curtains			Isothermal	34 °C
Baffles			Adiabatic	0
Columns			Adiabatic	0
Walls between columns			Adiabatic	0
Doors			Isothermal	32 °C
Drive-through alley floor			Isothermal	27 °C
Feed alley floor			Isothermal	27.5 °C
Compost bedded			Variable temperature	0.0836d+25.4 °C *
Service floor			Isothermal	28 °C
Fans support wall			Isothermal	30 °C
Laterals of pad cooling			Adiabatic	0
Pad cooling input	Opening	Entrainment: 95,320 Pa	Isothermal	29.7 °C
Fans	Outlet	Normal speed: 1.2 m s${}^{-1}$	-	-

* *d* is the distance from the cooling pads in meters.

**Table 2:** Statistics of the psychometric properties of the air measured at inlet and outlet of the cooling pad of the compost bedded dairy barn. The statistics of the predicted outlet conditions are shown between parenthesis $\bar{\mu }$ is the mean, $\tilde{\mu }$ is the median and σ is the standard deviation.

	Dry-Bulb Temperature			Relative Humidity		
	$\bar{\mu }$ [°C]	$\tilde{\mu }$ [°C]	σ [°C]	$\bar{\mu }$ [%]	$\tilde{\mu }$ [%]	σ [%]
Inlet	29.7	29.9	1.1	57.8	56.2	4.1
Outlet	24.4	24.4	0.7	82.1	81.8	2.4
	(24.6)	(24.7)	(0.7)	(82.1)	(81.4)	(2.6)

**Table 3:** Properties of the meshes analysed.

Mesh Properties		Mesh Sizes			
		Coarse	Medium	Fine${}_{1}$	Fine${}_{2}$
Number of elements		331,386	796,694	1,358,107	2,989,746
Number of nodes		1,048,893	2,675,770	4,655,442	10,236,714
Maximum element size		0.4 m	0.25 m	0.2 m	0.15 m
Skewness	Average	0.19631	0.14428	0.11884	0.10174
	Standard deviation	0.23201	0.20251	0.18236	0.16896
Orthogonal quality	Average	0.81062	0.86484	0.8893	0.90426
	Standard deviation	0.23101	0.19997	0.18057	0.16633
Aspect ratio	Average	22.041	10.007	6.4221	3.6717
	Standard deviation	44.33	30.339	23.628	16.129

**Table 4:** Statistics of psychometric properties of the air measured (*m*) outside and inside the tunnel-ventilated compost bedded dairy barn used to validate the proposed CFD model and the predictions made with it (*p*). $T_{db}$ is the dry-bulb temperature ([°C]), ϕ is the relative humidity ([%]), ETIC is the Equivalent Temperature Index for dairy Cattle ([°C]), *u* is the air velocity ([m s${}^{-1}$]), $\bar{\mu }$ is the mean, $\tilde{\mu }$ is the median and σ is the standard deviation.

	Hour Interval	Outside			Inside					
		${\bar{\mu }}_{m}$	${\tilde{\mu }}_{m}$	$\sigma_{m}$	${\bar{\mu }}_{m}$	${\bar{\mu }}_{p}$	${\tilde{\mu }}_{m}$	${\tilde{\mu }}_{p}$	$\sigma_{m}$	$\sigma_{p}$
$T_{db}$	10–11	27.5	27.4	0.2	26.3	25.4	26.4	25.4	0.8	0.6
	12–13	29.6	29.5	0.3	28.2	26.0	28.4	26.1	1.1	0.5
	14–15	32.2	32.3	0.4	28.9	27.1	29.3	27.2	1.4	0.5
	16–17	33.0	33.0	0.2	29.0	27.2	29.0	27.2	1.5	0.5
ϕ	10-11	72.8	73.0	1.6	74.1	84.9	73.5	84.3	6.5	2.3
	12–13	62.6	62.5	1.2	67.3	80.5	68.0	79.9	7.2	2.4
	14–15	52.7	52.6	1.9	62.3	75.0	63.0	74.7	6.1	1.9
	16–17	47.5	47.4	1.6	59.8	71.2	61.0	71.0	7.5	1.8
ETIC *	10-11	24.6	24.6	0.1	21.8	22.0	21.9	22.2	0.7	0.6
	12–13	25.1	25.3	1.0	23.1	22.2	22.8	22.4	0.9	0.5
	14–15	26.1	26.4	1.2	23.3	22.8	23.2	23.0	1.1	0.4
	15–16	26.4	26.4	0.2	23.0	22.5	23.0	22.6	1.0	0.4
*u*	-	-	-	-	1.4	1.3	1.4	1.3	0.1	0.2

* An air velocity of 0 m s^−1^ were considered for compute ETIC for the outside conditions.

**Figure A1:**
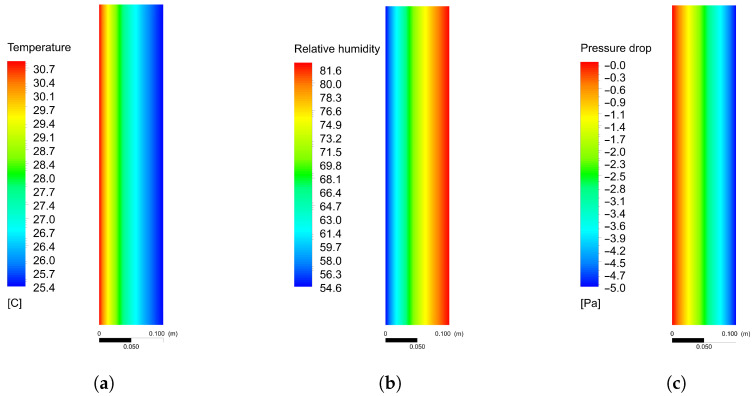
(**a**) Dry-bulb temperature [°C], (**b**) relative humidity [%] and (**c**) pressure drop ([Pa]) across the cellulose pad, predicted with the CFD model. For 30.9 °C, 54.5% and 1.2 m s${}^{-1}$ of dry-bulb temperature, relative humidity and velocity of the air at pad inlet, respectively.

**Figure A2:**
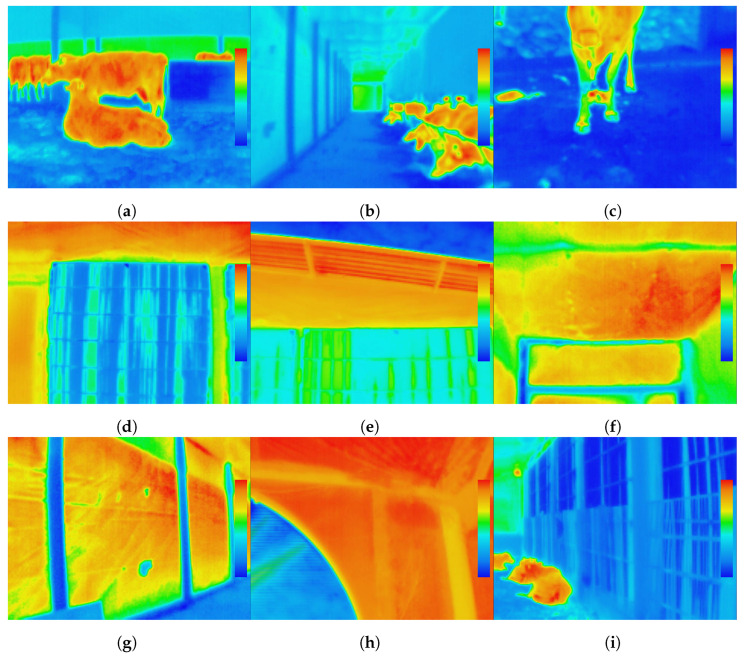
Examples of thermal images of the tunnel-ventilated compost-bedded pack barn analyzed. (**a**)—Compost-bedded pack. (**b**)—Drive-through feed alley. (**c**)—Feed alley. (**d**,**e**)—Exterior. (**f**)—False ceiling. (**g**)— Lateral curtain. (**h**)—Exhaust fans wall. (**i**)—Cooling pads.

## Data Availability

The data presented in this study are available on request from the corresponding author.

## Appendix A. Equations Used in the CFD Models

Due that the ANSYS CFX software solves the transport equations of the water vapour and the dry air in terms of the mass fraction of each one, the relative humidity (ϕ) of the inlet air has to be converted to water vapour mass fraction $m_{f_{w}}$. For this, the molar fraction of the water vapour ($M_{f_{w}}$) and the dry air ($M_{f_{a}}=1-M_{f_{w}}$) are computed using the Equation (A1) and replaced in the Equation (A2), where $M_{w_{w}}=18.01528$ g mol${}^{-1}$ and $M_{w_{a}}=28.966$ g mol${}^{-1}$ are the molar weights of the water and dry air, respectively.

(A1) $$M_{f_{w}}=\frac{\phi }{100}\frac{P_{v_{sat}}}{P_{T}}$$

(A2) $$m_{f_{w}}=M_{f_{w}}\frac{M_{w_{w}}}{M_{f_{w}}M_{w_{w}}+M_{f_{a}}M_{w_{a}}}$$

where $P_{T}$ is the atmospheric pressure in kPa and $P_{v_{sat}}$ is the saturation vapour pressure compute using the Arden Buck Equation (A3).

(A3) $$P_{v_{sat}}=0.61121e^{\left(18.678-\frac{T_{db}}{234.5}\right)\left(\frac{T_{db}}{257.14+T_{db}}\right)}\;\left[\mathrm{kPa}\right]$$

where $T_{db}$ is the dry-bulb temperature in degree Celsius.

Inversely, the Equation (A4) is used to convert the mass fraction of water vapour to relative humidity.

(A4) $$\phi =\frac{100}{1+\frac{m_{f_{a}}}{m_{f_{w}}}\frac{M_{w_{w}}}{M_{w_{a}}}}\;\left[\%\right]$$

The wet-bulb temperature ($T_{wb}$) is compute using the Equation (A5) proposed by [45].

(A5) $$\begin{array}{c} \begin{array}{cc} T_{wb}= & T_{db}\tan^{-1}\left(0.151977{\left(\phi +8.313659\right)}^{0.5}\right)+\tan^{-1}\left(T_{db}+\phi \right)-\\ & \tan^{-1}\left(\phi -1.676331\right)+0.00391838\phi^{1.5}\tan^{-1}\left(0.023101\phi \right)-4.686035\;[{}^{\circ}C] \end{array} \end{array}$$

The Reynolds (Re), Prandtl (Pr) and Schmidt (Sc) dimensionless numbers used to compute the heat and mass transfer coefficients are computed using the Equations (A6)–(A8).

(A6) $$Re=\frac{ul_{e}}{\nu }$$

(A7) $$Pr=\frac{\mu C_{p}}{k}$$

(A8) $$Sc=\frac{\nu }{D_{AB}}$$

where *u* is the air velocity [m s${}^{-1}$], $l_{e}$ is the characteristic length [m${}^{-1}$] of the cooling pad equal to reciprocal of the specific surface area, ν is the kinematic viscosity [m${}^{2}$ s${}^{-1}$], μ is the dynamic viscosity [kg m${}^{-1}$ s${}^{-1}$], $C_{p}$ is the specific heats of air [J kg${}^{-1}$ K${}^{-1}$], *k* is the thermal conductivity of air [W m${}^{-1}$ k${}^{-1}$] and $D_{AB}$ is the water vapour-air mass diffusivity coefficient [m${}^{2}$ s${}^{-1}$].

A third order polynomial were fitted (Equations (A9) to (A11), r${}^{2}$ = 1) to the data presented in the Table A-9 of [46] to compute ν, μ and *k* as function of the air temperature (*T* in degree Celsius).

(A9) $$\nu \left(T\right)=-3.574\times 10^{-13}T^{3}+1.284\times 10^{-11}T^{2}+8.664\times 10^{-8}T+1.338\times 10^{-5}$$

(A10) $$\mu \left(T\right)=3.263\times 10^{-13}T^{3}-6.690\times 10^{-11}T^{2}+4.933\times 10^{-8}T+1.729\times 10^{-5}$$

(A11) $$k\left(T\right)=-4.196\times 10^{-10}T^{3}+8.159\times 10^{-9}T^{2}+7.489\times 10^{-5}T+2.364\times 10^{-2}$$

The coefficients presented in the Table A-2 of [47] were used to form a third order polynomial to compute $C_{p}$ as function of the temperature (Equation (A12)).

(A12) $$C_{p}\left(T\right)=\frac{-1.0966\times 10^{-9}{\left(T+273.15\right)}^{3}+0.4802\times 10^{-5}{\left(T+273.15\right)}^{2}+0.1967\times 10^{-2}\left(T+273.15\right)+28.11}{2.8964\times 10^{-2}}$$

$D_{AB}$ was computed using the Equation (A13) presented in [48]

(A13) $$D_{AB}\left(T\right)=\frac{92.6\times 10^{-7}}{P_{T}}\frac{{\left(T-273.15\right)}^{2.5}}{\left(T-28.15\right)}$$

A third order polynomial were fitted (Equation (A14), R${}^{2}$ = 1) to the data presented in the Table A-4 of [47], to compute the water vapour density ($\rho_{v}\left[kgm^{-1}\right]$) as function of the air temperature.

(A14) $$\rho_{v}\left(T\right)=4.585\times 10^{-7}T^{3}-1.360\times 10^{-6}T^{2}+4.991\times 10^{-4}T+4.196\times 10^{-3}$$

## Appendix C. Spatial Distribution of Simulated Cows as Point Heat Sources

The spheres that represent the source points of convective heat loss from the cows, without mass and without presenting any resistance in the air flow was spatially random distributed according to the Cartesian coordinates given in the Table A1. The *x*-coordinate is measure from the drive-through feed alley to the service alley, and the *y*-coordinate is measure from the pad coolings to the fans negatively.

**Table A1:** Cartesian coordinates of the spatially random distributed spheres that represents the source points of convective heat loss from the cows, without mass and without presenting any resistance in the air flow.

Point	*x*	*y*	Point	*x*	*y*	Point	*x*	*y*	Point	*x*	*y*
1	11.3	−18.5	21	12.7	−12.5	41	15.1	−34.3	61	15.8	−51.8
2	14.6	−22.2	22	14.4	−2.1	42	5.5	−41.1	62	10.3	−49.2
3	16.3	−7.7	23	12.7	−2.6	43	12.4	−32.8	63	14.9	−54.0
4	9.6	−15.7	24	15.5	−3.2	44	12.5	−31.9	64	15.3	−49.2
5	14.6	−10.3	25	12.4	−15.9	45	17.8	−37.7	65	11.1	−41.5
6	10.7	−19.5	26	19.9	−11.6	46	11.8	−27.4	66	4.3	−42.9
7	17.5	−18.0	27	7.5	−4.4	47	10.3	−32.3	67	9.3	−53.1
8	17.3	−3.9	28	5.7	−11.6	48	14.7	−39.5	68	10.8	−47.9
9	8.1	−20.1	29	5.8	−3.5	49	15.9	−33.6	69	8.3	−52.9
10	13.8	−23.1	30	5.0	−1.3	50	12.3	−38.5	70	7.2	−44.1
11	13.3	−12.0	31	10.5	−19.9	51	9.6	−36.6	71	17.1	−48.8
12	12.7	−20.7	32	11.2	−13.1	52	6.4	−33.9	72	10.9	−49.9
13	17.9	−13.7	33	9.9	−21.7	53	13.4	−27.8	73	18.2	−41.7
14	8.2	−3.6	34	16.2	−16.3	54	8.2	−35.3	74	10.3	−49.7
15	9.1	−4.7	35	14.0	−33.8	55	4.7	−24.9	75	18.3	−46.2
16	5.9	−9.5	36	16.4	−38.0	56	16.1	−34.6	76	10.3	−41.9
17	19.0	−17.5	37	18.9	−39.1	57	7.9	−35.2	77	16.9	−48.0
18	14.3	−19.3	38	19.6	−41.1	58	11.1	−36.4	78	16.1	−43.9
19	11.7	−18.5	39	7.1	−23.4	59	15.0	−39.3	79	10.0	−42.9
20	14.2	−7.4	40	6.2	−38.8	60	9.7	−40.9	80	7.5	−44.1
